# Optimization of Regioselective α-Glucosylation of Hesperetin Catalyzed by Cyclodextrin Glucanotransferase

**DOI:** 10.3390/molecules23112885

**Published:** 2018-11-05

**Authors:** José L. González-Alfonso, Noa Míguez, J. Daniel Padilla, Laura Leemans, Ana Poveda, Jesús Jiménez-Barbero, Antonio O. Ballesteros, Georgina Sandoval, Francisco J. Plou

**Affiliations:** 1 Instituto de Catálisis y Petroleoquímica, CSIC, 28049 Madrid, Spain; josel.g@csic.es (J.L.G.-A.); noa.miguez@csic.es (N.M.); lauraleetin@gmail.com (L.L.); a.ballesteros@icp.csic.es (A.O.B.); gsandoval@ciatej.mx (G.S.); 2 Centro de Investigación y Asistencia en Tecnología y Diseño del Estado de Jalisco (CIATEJ), Guadalajara 44270, Jalisco, Mexico; jdpadilla@ciatej.mx; 3 Center for Cooperative Research in Biosciences, Parque Científico Tecnológico de Bizkaia, 48160 Derio, Biscay, Spain; apoveda@cicbiogune.es (A.P.); jjbarbero@cicbiogune.es (J.J.-B.)

**Keywords:** polyphenols, glycosylation, flavonoids, flavanones, cyclodextrin glucosyltransferase, enzymatic glucosylation, hesperidin, hesperetin

## Abstract

The regioselective α-glucosylation of hesperetin was achieved by a transglycosylation reaction catalyzed by cyclodextrin glucanotransferase (CGTase) from *Thermoanaerobacter* sp. using soluble starch as glucosyl donor. By combining mass spectrometry (ESI-TOF) and 2D-NMR analysis, the main monoglucosylated derivative was fully characterized (hesperetin 7-*O*-α-d-glucopyranoside). In order to increase the yield of monoglucoside, several reaction parameters were optimized: Nature and percentage of cosolvent, composition of the aqueous phase, glucosyl donor, temperature, and the concentrations of hesperetin and soluble starch. Under the optimal conditions, which included the presence of 30% of bis(2-methoxyethyl) ether as cosolvent, the maximum concentration of monoglucoside was approximately 2 mM, obtained after 24 h of reaction. To our knowledge, this is the first report of direct glucosylation of hesperetin employing free enzymes instead of whole cells.

## 1. Introduction

Dietary flavonoids (formerly called vitamin P) exhibit an ample range of biological activities, including antimicrobial, antihypertensive, antitumoral, neuroprotective and anti-inflammatory [1,2,3,4,5]. Among them, hesperetin (5,7,3′-trihydroxyl-4′-methoxylflavanone), which is mostly present in citrus fruits but also in tomatoes, apples and some flowers, has obtained notable interest due to its outstanding multifunctional medicinal properties [6,7]. On the basis of its ability to promote cellular antioxidant defense, hesperetin has been demonstrated to prevent the development of some chronic diseases including various types of cancer and Alzheimer’s disease [3,8,9,10]. Curiously, hesperetin also presents activity against parasites from tropical diseases [6,11].

The hydrophobic nature of many bioflavonoids partially explains their limited absorption in vivo [12,13]. In this context, hesperetin barely dissolves in water, and this low aqueous solubility limits its further pharmacological exploitation. It is well reported that various flavonoids are glycosylated in nature and these sugar moieties play a major role in their absorption [14], bioavailability [15], bioactivity [16] and water solubility [17]. Glycosylation can also protect bioflavonoids from oxygen and/or light degradation, as well as during storage and gastrointestinal transit after ingestion [18].

Unfortunately, hesperidin (hesperetin 7-rutinoside), the natural glycosylated form of hesperetin, also exhibits a negligible solubility in water. Different glycosides of hesperidin have been synthesized in order to expand its biopharmaceutical applications and improve its bioavailability [19,20,21]. A Japanese company commercializes an α-glucosylated derivative of hesperidin with improved performance in cosmetic preparations [22].

For glycosylation of flavonoids, the use of enzymes is a very attractive strategy due to their unique specificity and the mild conditions required for such biotransformations [23,24,25,26,27]. The enzymatic glycosylation of hesperetin has been scarcely investigated, and only a few reports have been published employing cultured cells from yeasts or plants. In particular, Shimoda et al. achieved the α-glucosylation of hesperetin by the use of *Xanthomonas campestris* cells; the resulting monoglucosides were further enzymatically glycosylated into the corresponding maltosides [28]. In other work, the same group employed cultured cells of *Ipomoea batatas* and *Eucalyptus perriniana* to convert hesperetin into various mono- and di-β-glucosides [29]. An indirect way to obtain mono-β-glucosylated derivatives of hesperetin was developed by other authors, which relies on the controlled hydrolysis of hesperidin by α-rhamnosidase.

In this work, we describe the enzymatic synthesis of an α-glucosylated derivative of hesperetin by a transglycosylation reaction catalyzed by a transglycosidase, namely cyclodextrin glycosyltransferase (CGTase, EC 2.4.1.19), which employs a renewable and easily available carbohydrate (starch) as glucosyl donor [30]. In previous works, we successfully achieved the α-glucosylation of a flavonoid (epigallocatechin gallate [26]) and two stilbenes (resveratrol [31], pterostilbene [32]) with this enzyme.

## 2. Results and Discussion

### 2.1. Enzymatic Glucosylation of Hesperetin

We screened by TLC a series of glycosidases and transglycosidases for the glycosylation of racemic (±)-hesperetin (6 mg/mL), using the corresponding sugar donors (50 mg/mL). The screening was performed at 50 °C in presence of 30% (*v*/*v*) acetonitrile because the solubility of hesperetin in aqueous media is almost negligible. The aqueous phase was 10 mM sodium acetate (pH 5.6), an appropriate buffer for most of the glycosidic enzymes. Results were negative with most of the enzymes tested: β-galactosidases from *Bacillus circulans* and *Aspergillus oryzae* (sugar donor: Lactose); α-glucosidases from *Aspergillus niger* and *Xanthophyllomyces dendrorhous* (sugar donor: Maltose); β-fructofuranosidases from *Saccharomyces cerevisiae* and *X. dendrorhous* (sugar donor: Sucrose). The only enzyme that gave rise to an appreciable formation of glycosylated products was cyclodextrin glucanotransferase (CGTase) from *Thermoanaerobacter* sp. (Toruzyme 3.0L) [30,33], employing partially hydrolyzed starch (maltodextrins with DP ≤ 60) as glucosyl donor. This enzyme has demonstrated an extraordinary ability to glucosylate other mono- and polyphenolic compounds [26,31,32,34,35,36,37,38].

**Figure 1:**
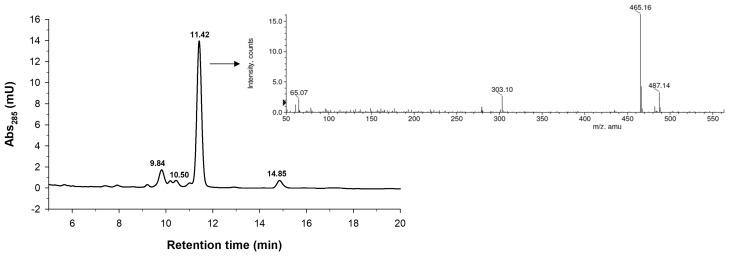
HPLC-MS chromatogram of the reaction mixture after 27 h showing the glucosides of hesperetin obtained with the CGTase from *Thermoanaerobacter* sp. Inset: Mass spectrum (ESI-TOF in positive mode) of the main peak at 11.42 min. Reaction conditions: Hesperetin (20 mg/mL), soluble starch (45 mg/mL), 10 mM sodium acetate buffer (pH 5.6), 35% acetonitrile, CGTase from *Thermoanaerobacter* sp. (5% *v*/*v*), 50 °C, 150 rpm.

The reaction mixture was characterized in detail by HPLC coupled to mass spectrometry (Figure 1). As shown, we detected the formation of several glucosides. The mass spectrum of the main peak at 11.42 min is shown in the inset of Figure 1. We observed the presence of peaks at *m*/*z* 465.16 and 487.14 that corresponded to the M + [H]^+^ and M + [Na]^+^ ions of the hesperetin monoglucoside, respectively. Based on HPLC/MS data, we concluded that the peaks with retention times of 10.50, 11.42 and 14.85 were monoglucosides, whilst the one that eluted at 9.84 min was a diglucoside. It cannot be ruled out that, in the diglucoside, the second glucosyl moiety could be attached to the first glucosyl residue by α(1→4) linkage. CGTase and related transglycosidases typically have a tendency to form homologous series of glucosylated products with increasing polymerization degree [39,40,41].

**Figure 2:**
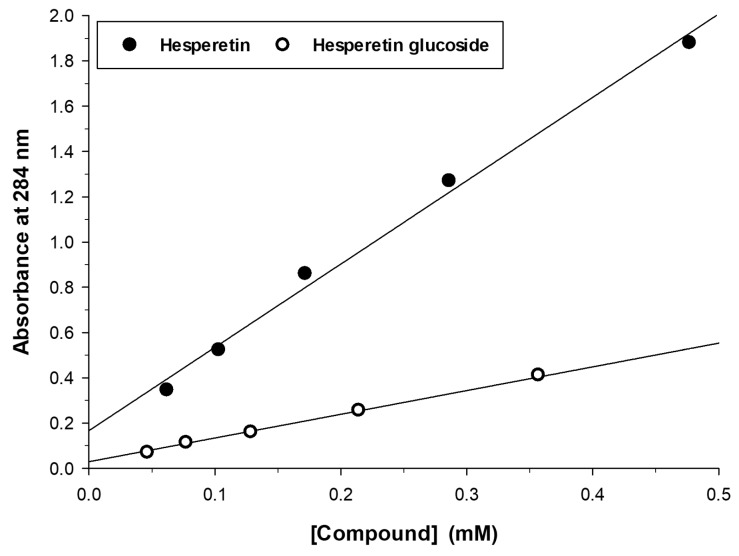
Absorbance at 284 nm vs. concentration for hesperetin and its monoglucoside.

The main glucoside of hesperetin (peak at 11.42 min) was purified by semipreparative HPLC. In order to quantify the production of this compound under different experimental conditions, we measured its extinction coefficient at 284 nm (Figure 2). Interestingly, the glucosylation altered the spectral properties of hesperetin, giving rise to a 3.5-fold reduction of the ε_284_.

### 2.2. Characterization of the Monoglucosylated Derivative

The high-resolution mass spectrum (HRMS) of the isolated glucoside obtained by electrospray (ESI-TOF) contained a pseudomolecular ion [M + Na]^+^ peak at *m*/*z* 487.1217 (Supplementary Materials, Figure S1), consistent with a molecular formula of C_22_H_24_O_11_ (calcd. 487.1230 for C_22_H_24_NaO_11_).

**Figure 3:**
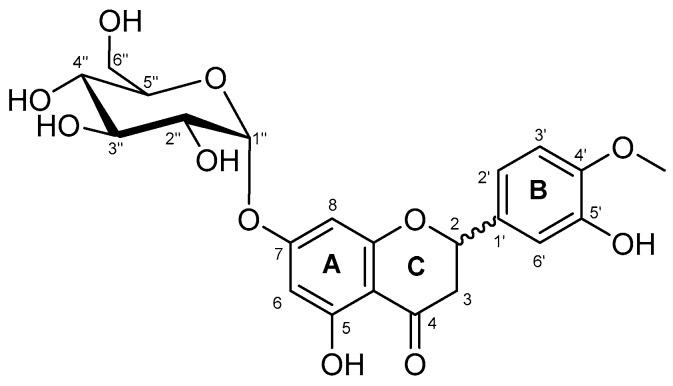
Chemical structure of hesperetin 7-*O*-α-d-glucopyranoside.

The structure of the glucosylated derivative was deduced by using 2D-NMR (see Table 1 and Figures S2–S7 of Supplementary Materials). The analysis of the NMR data permitted to show, in a non-ambiguous manner, that glucosylation of the hesperetin takes place with α-configuration at position O-7 of the A ring (Figure 3). Indeed, NOEs were observed between the anomeric H1′ proton and protons H6 and H8 of A ring. The α-configuration was deduced from the sugar ^3^*J*_H1–H2_ coupling value of 3.4 Hz. In addition, a correlation HMBC peak was observed between the same H1′ proton and C7 of A ring. The NMR spectroscopic data for hesperetin 7′-*O*-α-d-glucopyranoside is summarized in Table 1. These results fit well with the data reported by Li et al. who isolated hesperetin 7-*O*-α-d-glucopyranoside from the peel of citrus species [42]. Since the starting material is racemic [(±)-hesperetin], the final monoglucosylated product is a 50/50 mixture of diastereoisomers, due to the presence of epimers at carbon C-2. Thus, several signals appeared duplicated in the spectra.

The synthesis of this compound was previously reported by Shimoda et al. using Xanthomonas campestris cells [28], who obtained a mixture of 3′-, 5- and 7-*O*-α-d-glucosides. However, no spectroscopy data of such compounds are available in that work. Other articles dealing with glucosylation of hesperetin described the formation of β-glucosides, employing plant cultured cells of Ipomoea batatas and Eucalyptus perriniana [29], and Citrus paradisi [43]. To our knowledge, our present work describes for the first time the glucosylation of hesperetin employing free enzymes instead of whole cells.

### 2.3. Optimization of Hesperetin Glucosylation

**Figure 4:**
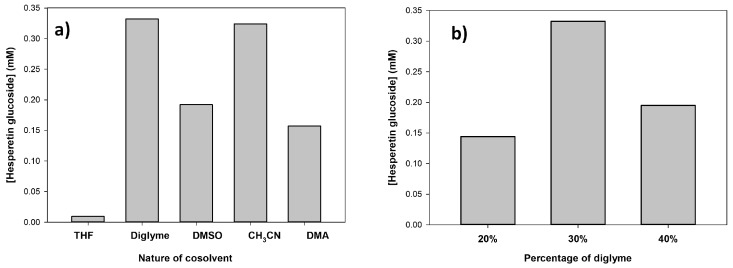
(**a**) Effect of different cosolvents at 30 % (*v*/*v*) on the glucosylation of hesperetin. (**b**) Effect of the concentration of bis(2-methoxyethyl) ether. Reaction conditions: hesperetin (6 mg/mL), partially hydrolyzed starch (50 mg/mL), CGTase from *Thermoanaerobacter* sp. (10% *v*/*v*), 10 mM acetate buffer pH 5.6 (50–70%), organic solvent (20–40%), 50 °C, 1000 rpm. The reactions were analyzed after 18 h by HPLC as described.

Several reaction parameters were assessed in order to increase the yield of the hesperetin monoglucoside. First, the effect of the nature of the organic cosolvent was studied fixing the organic solvent concentration at 30% *v*/*v*. Figure 4a represents the concentration of the 7′-*O*-α-d-glucoside after 18 h reaction. As shown, bis(2-methoxyethyl) ether (diglyme) and acetonitrile gave rise to the highest production levels of the monoglucoside. No reaction was observed in absence of cosolvent and it was very slow in the case of THF. Then, we varied the percentage of diglyme in the range 20–40%. As illustrated in Figure 4b, 30% *v*/*v* was the optimum value. It is likely that increasing the cosolvent percentage above 30% could cause certain inactivation of CGTase, despite the enhancement of hesperetin solubility.

The transglycosylation activity of CGTase can be displayed by coupling or disproportionation reactions, which utilize cyclodextrins or maltodextrins, respectively, as glucosyl donors [44,45]. We compared the formation of hesperidin glucoside using α-CD or γ-CD (coupling activity) and soluble starch (disproportionation activity) as glucosyl donors using 30% *v*/*v* diglyme as cosolvent. We concluded that partially hydrolyzed starch was the most efficient donor (data not shown).

We also analyzed the effect of the composition of the aqueous phase; we assessed different buffers (10 mM sodium citrate pH 5.0, 10 mM sodium acetate pH 5.6) and water. Citrate buffer pH 5.0 gave rise to the highest yield of monoglucoside (data not shown).

**Figure 5:**
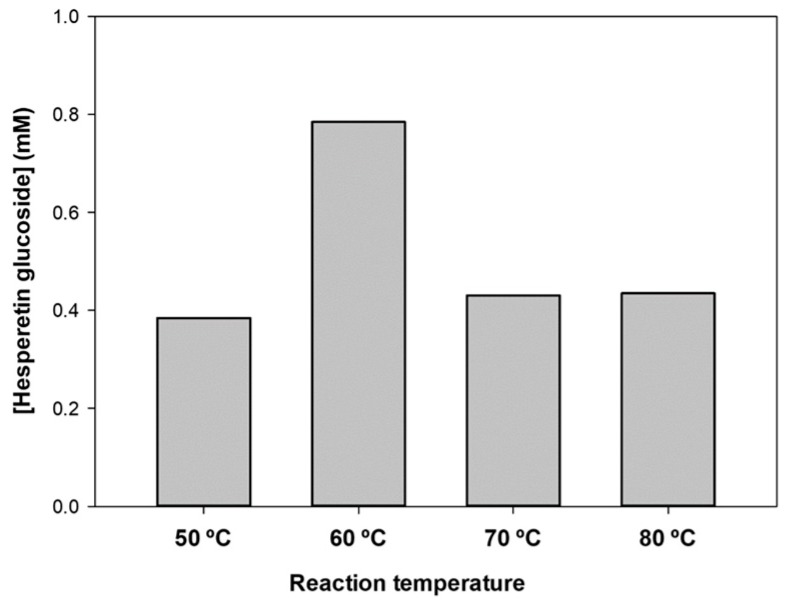
Effect of temperature on the glucosylation of hesperetin. Reaction conditions: hesperetin (6 mg/mL), soluble starch (50 mg/mL), CGTase from *Thermoanaerobacter* sp. (10% *v*/*v*), sodium citrate 10 mM at pH 5.0 (60% *v*/*v*), bis(2-methoxyethyl) ether (30 % *v*/*v*), 1000 rpm. The reactions were analyzed after 18 h.

Then, we studied the effect of temperature on the reaction course. Figure 5 represents the formation of hesperetin monoglucoside at different temperatures (50–80 °C) after 18 h. As illustrated, 60 °C was the optimum temperature, in accordance with other biotransformations catalyzed by this enzyme [39,46], since this temperature represents a compromise between enzyme activity and protein stability.

**Figure 6:**
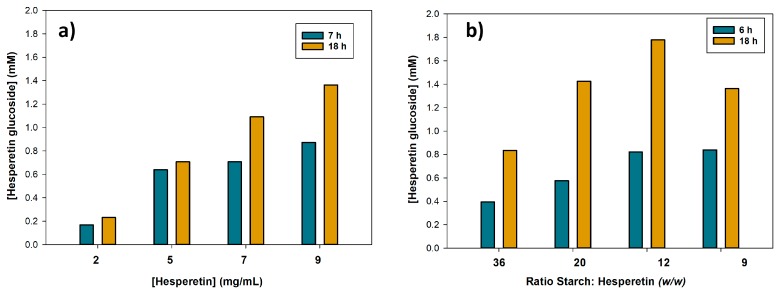
Effect of the concentrations of soluble starch and hesperetin on the glucosylation. (**a**) Maintaining a ratio 20:1 *w*/*w* starch/hesperetin; (**b**) Fixing the concentration of partially hydrolyzed starch (180 mg/mL) and varying the amount of hesperetin. Reaction conditions: hesperetin (2–20 mg/mL), soluble starch (24–180 mg/mL), CGTase from *Thermoanaerobacter* sp. (10% *v*/*v*), bis(2-methoxyethyl) ether (30 % *v*/*v*), 10 mM sodium citrate buffer pH 5.0 (60% *v*/*v*), 60 °C, 1000 rpm.

The ratio glucosyl donor: acceptor is a critical point in enzyme-catalyzed transglycosylations. First, we varied the concentrations of soluble starch and hesperetin but maintaining a ratio 20:1 *w*/*w* between them (Figure 6a). As shown, the higher the concentration of hesperetin and starch, the higher the amount of glucoside synthesized. In a second step, we fixed the concentration of soluble starch (180 mg/mL) and varied the concentration of hesperetin in the range 5–20 mg/mL (Figure 6b). The maximum yield of glucoside after 18 h reaction (1.8 mM) was obtained using 15 mg/mL hesperetin, which corresponded to a ratio 12:1 *w*/*w* starch/hesperetin.

### 2.4. Progress of Hesperetin Glucosylation under Optimal Conditions.

**Figure 7:**
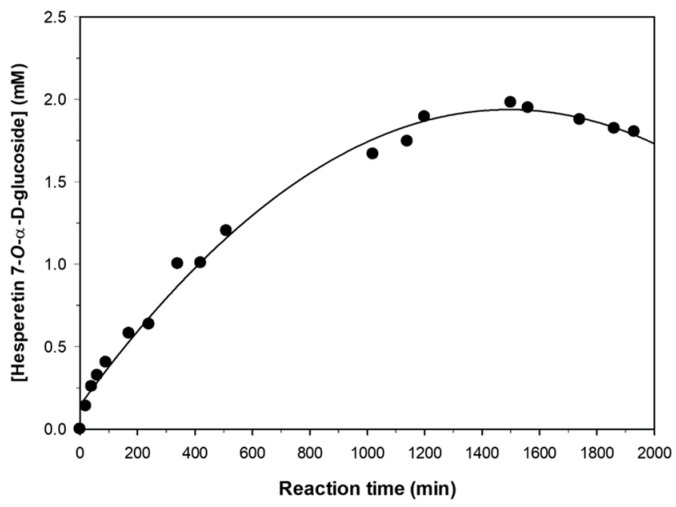
Progress of hesperetin glucosylation catalyzed by CGTase from *Thermoanaerobacter* sp. under the optimal conditions: hesperetin (15 mg/mL), soluble starch (180 mg/mL), CGTase (10% *v*/*v*), bis(2-methoxyethyl) ether (30% *v*/*v*), 10 mM sodium citrate buffer pH 5.0 (60% *v*/*v*), 60 °C, 1000 rpm.

We analyzed in detail the formation of hesperetin 7-*O*-α-d-glucopyranoside under the optimal conditions described before (Figure 7). As shown, the maximum concentration of monoglucoside was approximately 2 mM, obtained at 24 h reaction. Taking into account that the initial concentration of hesperetin was 49 mM (although the actual value was significantly much lower because most of the hesperetin remained in solid form, even in the presence of 30% *v*/*v* diglyme), the conversion yield was close to 4.1%. Shimoda et al. reported yields between 10–15% of the 3′-, 5- and 7-*O*-α-d-glucosides [28]; however, they employed a concentration of hesperetin of 0.02 mM, 2500-fold lower than the used in the present work. In addition, the enzymatic process carried out with CGTase is more regioselective than that mentioned before. In addition, the β-glucosylation of hesperetin catalyzed by plant cells also takes place with low selectivity [29,43].

After the point of maximum concentration (Figure 7), the monoglucoside was slowly hydrolyzed by the action of CGTase. This behavior is typically observed in kinetically-controlled transglycosylations catalyzed by glycosidic enzymes [47,48,49].

## 3. Materials and Methods

### 3.1. Chemicals

(±)-Hesperetin was from Sigma-Aldrich. Partially hydrolyzed starch from potato (Paselli SA2) was from Avebe (Foxhol, The Netherlands). All other reagents and solvents were of the highest available purity and used as purchased.

### 3.2. Enzymes

The cyclodextrin glucanotransferase (CGTase) from *Thermoanaerobacter* sp. (Toruzyme 3.0 L) was kindly provided by Novozymes A/S. Toruzyme 3.0 L was partially purified by a PD-10 desalting column (GE Healthcare, Chicago, IL, USA).

### 3.3. Optimal Conditions for Enzymatic Glucosylation of Hesperetin

Hesperetin (15 mg) and partially hydrolyzed starch (180 mg) were dissolved in a mixture of 10 mM sodium citrate buffer pH 5.0 (0.6 mL) and bis(2-methoxyethyl) ether (0.3 mL). Partially purified CGTase from *Thermoanaerobacter* sp. (0.1 mL) was then added to a final concentration of 10% *v*/*v*. The mixture was maintained at 60 °C in an orbital shaker (model SI50, Stuart Scientific, Staffordshire, UK) at 1000 rpm. Aliquots (150 μL) were withdrawn at intervals, diluted with 150 μL of acetonitrile to stop the reaction, passed through 0.45 μm nylon filters (Cosela, Sevilla, Spain) and analyzed by HPLC.

### 3.4. High-Performance Liquid Chromatography (HPLC)

HPLC analysis was performed using a quaternary pump (model 600, Waters, Milford, MA, USA) coupled to an autosampler (Varian ProStar, Palo Alto, CA, USA, model 420). The column was ACE 3 C18-PFP (4.6 × 150 mm, Symta, Madrid, Spain). The temperature of the column was kept constant at 40 °C. The injection volume was 20 µL. The detection of peaks was carried out using a photodiode array detector (ProStar, Varian, Palo Alto, CA, USA) and peaks were analyzed using the Varian Star LC workstation 6.41. Quantification was performed at 284 nm. The mobile phase was acetonitrile/water 60:40 (*v*/*v*) at 0.7 mL/min during 25 min. Both solvents contained 0.1% (*v*/*v*) of formic acid.

### 3.5. HPLC coupled to Mass Spectrometry (HPLC-MS)

The molecular weight of reaction products was determined by HPLC-MS using a HPLC 1100 (Agilent Technologies, Santa Clara, CA, USA) equipped with a photodiode array detector and coupled to mass spectrometer (Maxis II, Bruker, Billerica, MA, USA) with hybrid QTOF analyzer. Samples were ionized by electrospray (with nitrogen to desolvate the mobile phase) and analyzed in positive reflector mode. The analysis was performed with an ACE C18-PFP column (4.6 × 100 mm, Symta, Madrid, Spain). The mobile phase was MeOH/H_2_O 48:52 (*v*/*v*). Both solvents contained 0.1% (*v*/*v*) of formic acid. The flow rate was 0.7 mL/min, and the column temperature was kept constant at 40 °C.

### 3.6. Purification of the Main Hesperetin Glucoside

The glucosylation of hesperetin was scaled up. The reaction mixture contained hesperetin (180 mg), soluble starch (3.6 g), Toruzyme 3.0 L (2 mL), 14 mL of 10 mM sodium citrate buffer pH 5.0 and 6 mL of bis(2-methoxyethyl) ether. The mixture was incubated for 24 h at 60 °C with orbital shaking (model SI50, Stuart Scientific, Staffordshire, UK) at 150 rpm. The mixture was then cooled and concentrated, and the glucosylated derivatives of hesperetin were purified using semipreparative HPLC. The column was ACE 5 C18-PFP (10 × 250 mm, Symta). A three-way flow splitter at 1/10 (Accurate, LC Packings, SpectraLab, Ontario, ON, Canada) was employed. The starting mobile phase was MeOH/water 50:50 (*v*/*v*) and the gradient is described in Table 2. Both solvents contained 0.1% (*v*/*v*) of formic acid. The flow rate was 4.6 mL/min, and the column temperature was kept constant at 40 °C. After collecting the glucosylated products, the mobile phase was eliminated by rotatory evaporation in an R-210 rotavapor (Buchi, Essen, Germany). The isolated products were characterized by mass spectroscopy and NMR.

### 3.7. Mass Spectrometry (MS)

The HRMS of purified hesperetin monoglucoside was assessed using a mass spectrometer with hybrid QTOF analyzer (model QSTAR, Pulsar i, AB Sciex, Madrid, Spain). The sample was analyzed by direct infusion and ionized by electrospray (with methanol containing 1% of NH_4_OH as ionizing phase) in positive reflector mode.

### 3.8. Nuclear Magnetic Resonance (NMR)

The structure of the monoglucosylated hesperetin derivative was determined using a combination of 1D and 2D (COSY, DEPT-HSQC, NOESY) standard NMR techniques. The spectra of the sample, dissolved in DMSO-*d*_6_ (ca. 7 mM), were recorded on a Bruker IVDr 600 spectrometer equipped with a BBI probe with gradients in the *Z* axis, at a temperature of 300 K. Chemical shifts were expressed in parts per million (ppm). Residual DMSO-*d*_5_ signal was used as internal reference (2.5 ppm). All the employed pulse sequences were provided by Bruker. For the DEPT-HSQC experiment, values of 8 ppm and 1 K points, for the ^1^H dimension, and 165 ppm and 256 points for the ^13^C dimension, were used. For the homonuclear COSY and NOESY experiments, 8 ppm windows were used with a 1 K × 256 point matrix. For the NOESY the mixing time was 500 ms.

## 4. Conclusions

We report for the first time a regioselective enzymatic process for the glucosylation of hesperetin. The glucosyl moiety is attached to the flavonoid in α-configuration. Previous works of hesperetin glycosylation were carried out with whole cells and, except in one case [28], the resulting products were β-glucosides. Glycosylation of hesperetin has been also achieved by hesperidin hydrolysis catalyzed by α-rhamnosidases [50], but in this case the glucosides present β-configuration. We demonstrated in a previous work on glucosylation of resveratrol that the anomeric configuration of the linked glucose exerts a substantial effect on the physico-chemical and surfactant properties of the synthesized products [31]. The synthesized monoglucoside could be of interest in the nutraceutical, cosmetic and pharmaceutical industries, as occurs with hesperidin glucosides [51]. Nevertheless, further studies on its bioavailability and bioactivity are necessary to estimate its full potential [52].

## Figures and Tables

**Table 1:** NMR Spectroscopic Data (600 MHz, DMSO-*d*_6_) for hesperetin 7-*O*-α-d-glucopyranoside. Data obtained from HSQC and HMBC spectra.

Position	δ_H_	δ_C_
2	5.48	78.2
3	2.8, 3.3	42.1
4	-	196.8
5 ^a^	-	162.6
6 ^b^	6.2 ^c^	95.6
7	-	164.7
8 ^b^	6.18	96.5
9 ^a^	-	162.2
10	-	103.0
1′	-	130.5
2′	6.9 ^c^	117.5
3′	6.9	111.7
4′	-	147.6
5′	-	146.1
6′	6.9	113.8
MeO	3.8	55.4
1′′	5.50, 5.53 (*J* = 3.4 Hz) ^c^	96.7
2′′	3.4	71
3′′	3.6	72.7
4′′	3.2	69.4
5′′	3.3	73.9
6′′	3.54, 3.46	60.2
OH2′′	5.1 ^c^	-
OH3′′	4.97	-
OH4′′	5.0	-
OH6′′	4.5	-

^a^ These ^13^C assignations can be interchanged; ^b^ These ^1^H and ^13^C assignations can be interchanged; ^c^ Duplicated signals due to the presence of diasteroisomers.

**Table 2:** HPLC gradient profile for purification of hesperetin monoglucoside.

Time	Methanol (%)	Water (%)
0–2 min	50	50
2–5 min	52	48
5–10 min	55	45
10–20 min	60	40
20–30 min	70	30

## Supplementary Materials

The following figures are available online, Figure S1: HRMS spectrum for hesperetin 7′-*O*-α-d-glucopyranoside; Figure S2: NMR Spectra (600 MHz, DMSO-*d*_6_, 298 K) for hesperetin and its 4′-*O*-α-d-glucopyranoside; Figure S3: ^1^H–^13^C HSQC edited NMR Spectrum (600 MHz, DMSO-*d*_6_, 298 K) for hesperetin 7′-*O*-α-d-glucopyranoside; Figure S4: COSY NMR Spectrum (600 MHz, DMSO-*d*_6_, 298 K) for hesperetin 7′-*O*-α-d-glucopyranoside; Figure S5: NOESY NMR Spectrum (600 MHz, DMSO-*d*_6_, 298 K) for hesperetin 7′-*O*-α-d-glucopyranoside; Figure S6: ^1^H-^13^C HMBC NMR Spectrum (600 MHz, DMSO-*d*_6_, 298 K) for hesperetin 7′-*O*-α-d-glucopyranoside; Figure S7: Hesperetin (up) and hesperetin 7′-*O*-α-d-glucopyranoside signals affected by the diastereoisomers formation (down).
