# Supplementary material for: Optimization of Regioselective α-Glucosylation of Hesperetin Catalyzed by Cyclodextrin Glucanotransferase
Source: Molecules. 2018 Nov 5;23(11):2885. doi: 10.3390/molecules23112885 (PMC6278433; doi:10.3390/molecules23112885)

# Optimization of regioselective $\alpha$-glucosylation of hesperetin catalyzed by cyclodextrin glucanotransferase

**José L. González-Alfonso[1], Noa Míguez[1], J. Daniel Padilla[2], Laura Leemans[1], Ana Poveda[3], Jesús Jimnez-Barbero[3], Antonio O. Ballesteros[1], Georgina Sandoval[2] and Francisco J. Plou[1,*]**

[1] Instituto de Catálisis y Petroleoquímica, CSIC, 28049 Madrid, Spain; josel.g@csic.es (J.L.G.-A.); noa.miguez@csic.es (N.M.); lauraleetin@gmail.com (L.L.); a.ballesteros@icp.csic.es (A.O.B.)
[2] Unidad de Biotecnología Industrial, Centro de Investigación y Asistencia en Tecnología y Diseño del Estado de Jalisco (CIATEJ), Guadalajara, Jalisco (44270), México; jdpadilla@ciatej.mx (J.D.P.); gsandoval@ciatej.mx (G.S.)
[3] Center for Cooperative Research in Biosciences, Parque Científico Tecnológico de Bizkaia, 48160 Derio, Biscay, Spain; apoveda@cicbiogune.es (A.P); jjbarbero@cicbiogune.es (J.J.-B)

* Correspondence: fplou@icp.csic.es; Tel.: +34-91-585-4869

**Figure S1:** HRMS spectrum for hesperetin 7′-O-$\alpha$-D-glucopyranoside.

**Figure S2:** NMR Spectra (600 MHz, DMSO-d6, 298 K) for hesperetin 7′-O-$\alpha$-D-glucopyranoside.

**Figure S3:** $^{1}$H-$^{13}$C HSQC edited NMR Spectrum (600 MHz, DMSO-d6, 298 K) for hesperetin 7′-O-$\alpha$-D-glucopyranoside.

**Figure S4:** COSY NMR Spectrum (600 MHz, DMSO-d6, 298 K) for hesperetin 7′-O-$\alpha$-D-glucopyranoside.

**Figure S5:** NOESY NMR Spectrum (600 MHz, DMSO-d6, 298 K) for hesperetin 7′-O-$\alpha$-D-glucopyranoside.

**Figure S6:** $^{1}$H-$^{13}$C HMBC NMR Spectrum (600 MHz, DMSO-d6, 298 K) for hesperetin 7′-O-$\alpha$-D-glucopyranoside.

**Figure S7:** Hesperetin (up) and hesperetin 7′-O-$\alpha$-D-glucopyranoside signals affected by the diastereoisomers formation (down)

**Figure S1: HRMS spectrum for hesperetin 7′-O-$\alpha$-D-glucopyranoside.**

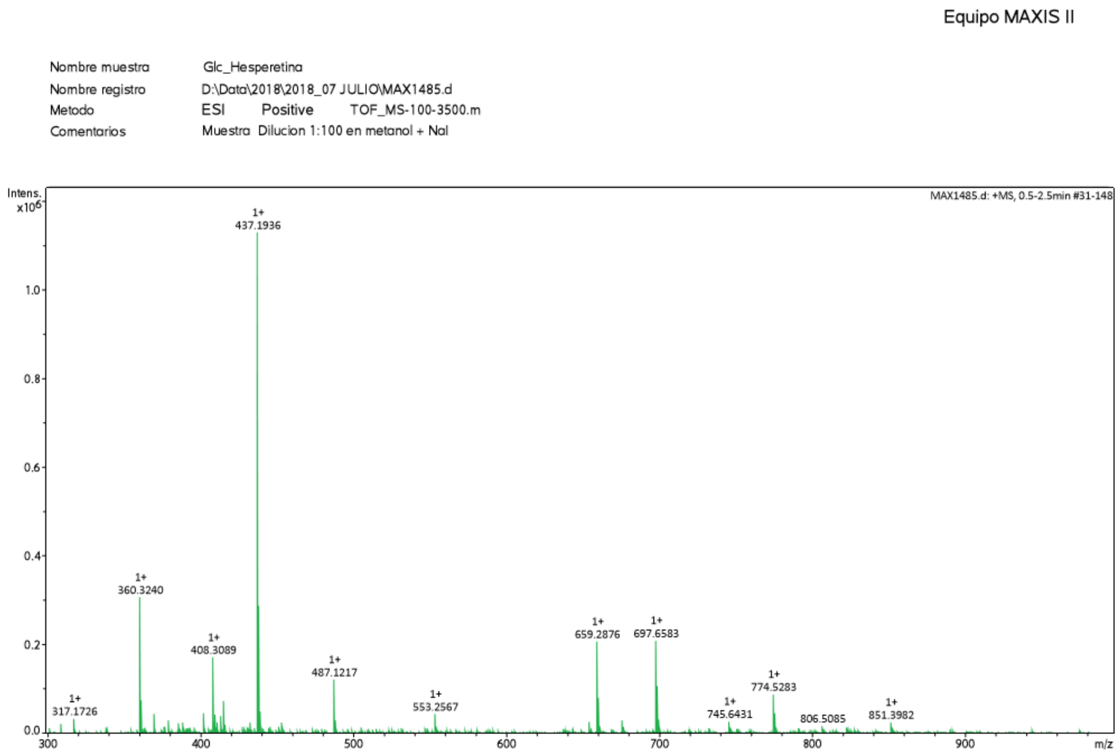

**Figure S2: ¹H NMR Spectrum (600 MHz, DMSO-d6, 298 K) for hesperetin 7′-O-α-D-glucopyranoside.**

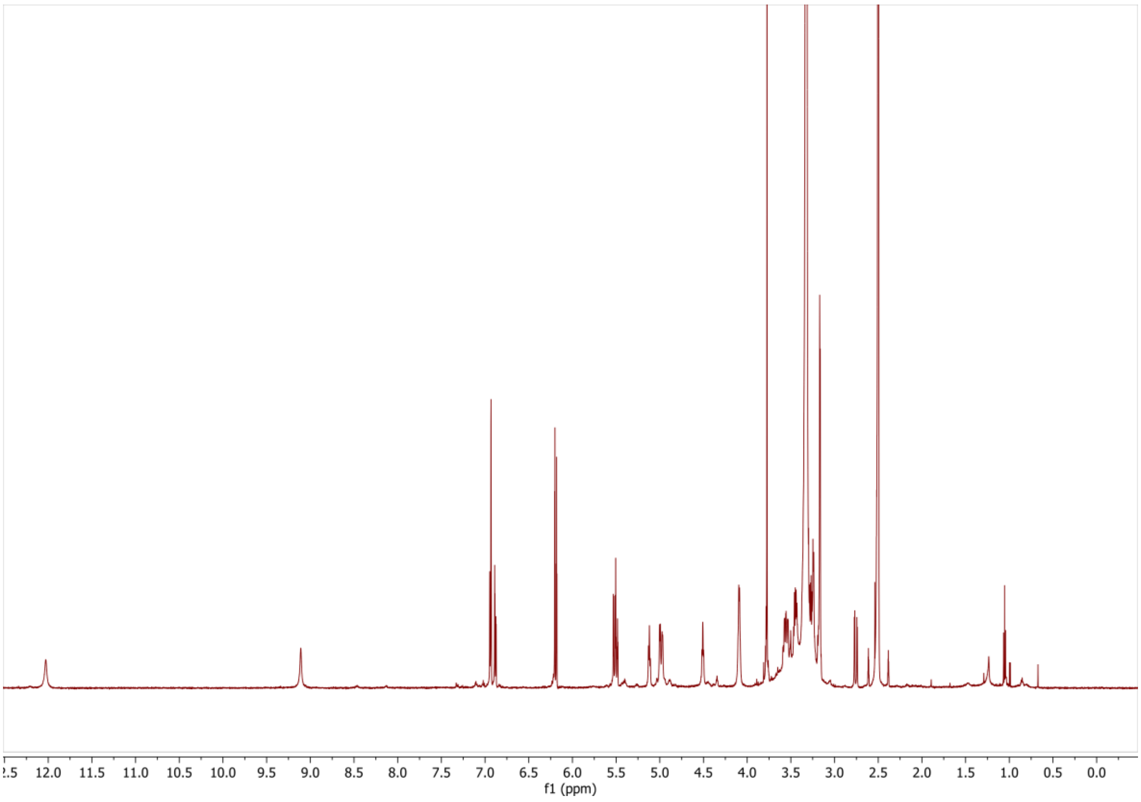


**Figure S3: ¹H-¹³C HSQC edited NMR Spectrum (600 MHz, DMSO-d6, 298 K) for hesperetin 7′-O-α-D-glucopyranoside. Some impurities are shown**

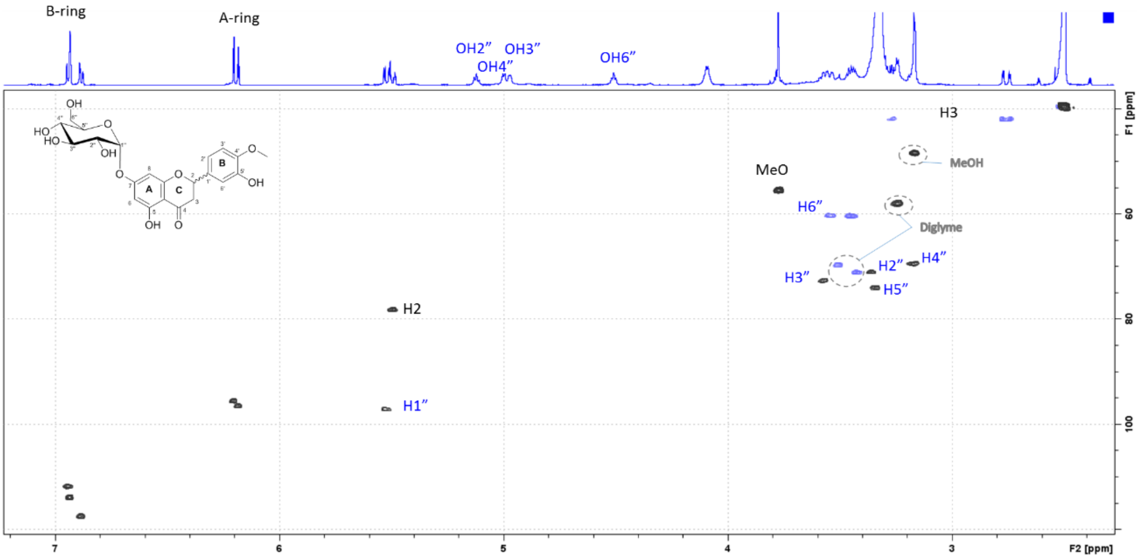

**Figure S4: COSY NMR Spectrum (600 MHz, DMSO-d6, 298 K) for hesperetin 7′-O-α-D-glucopyranoside.**

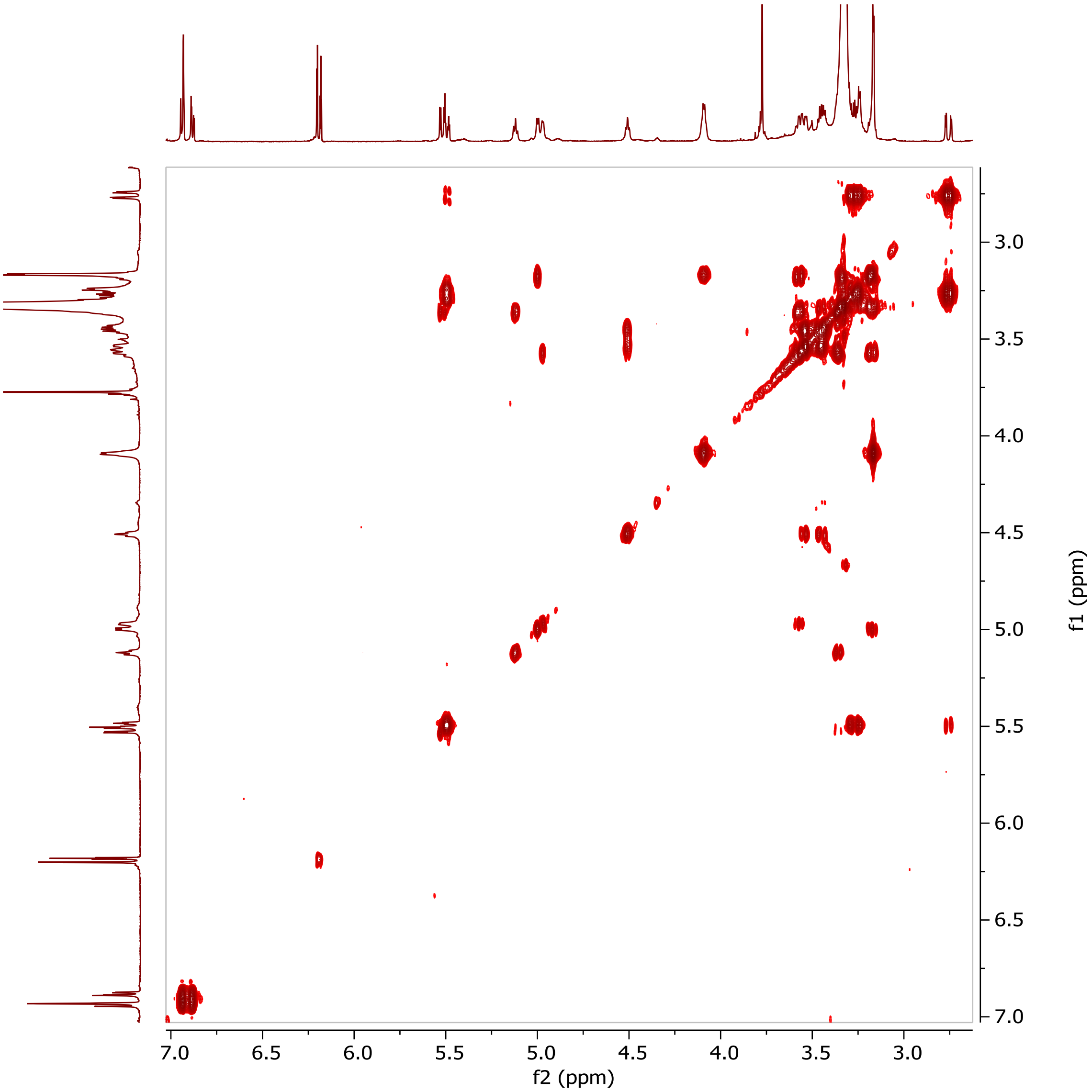

**Figure S5: NOESY NMR Spectrum (600 MHz, DMSO-d6, 298 K) for hesperetin 7′-O-α-D-glucopyranoside.**

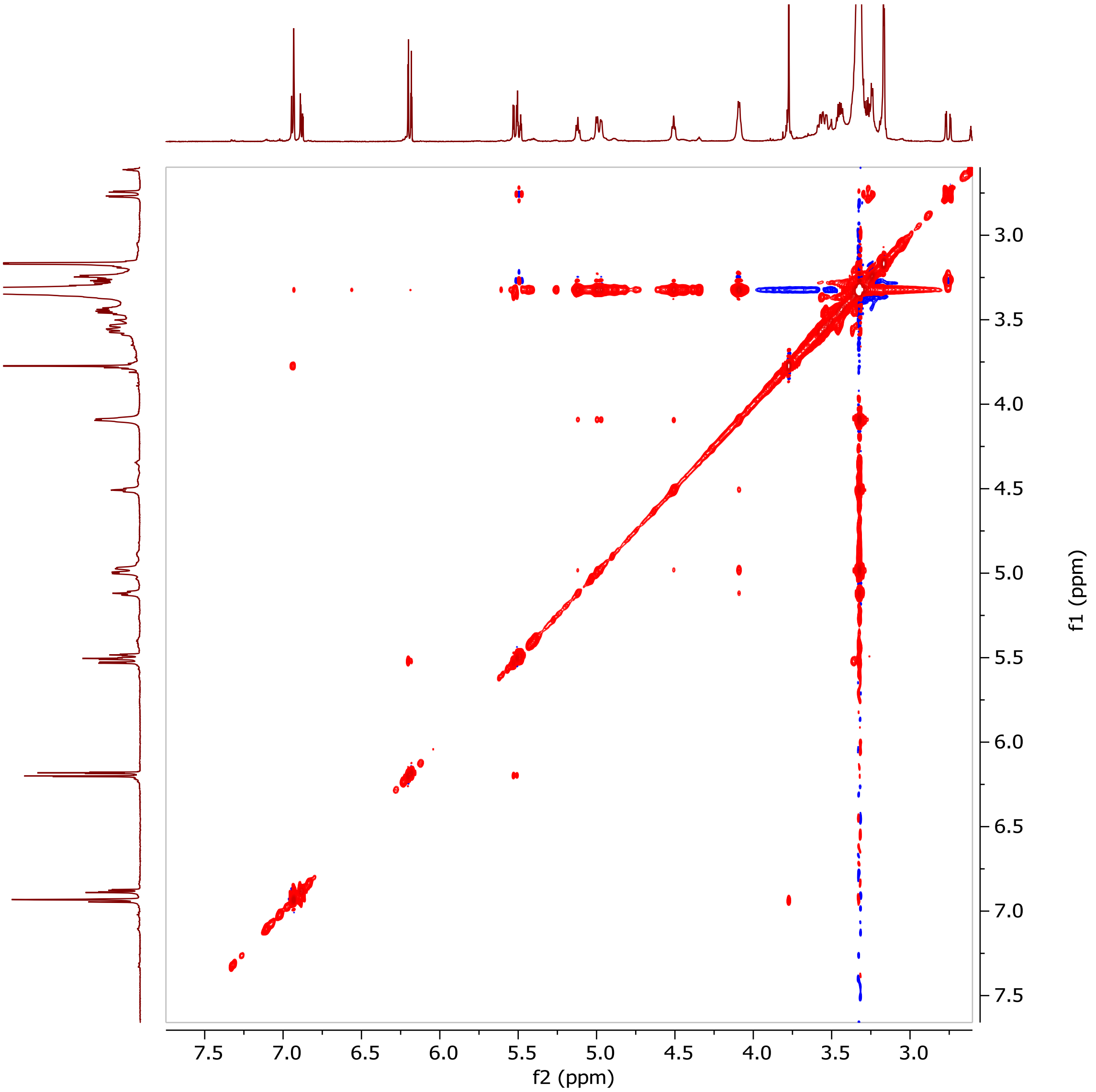

**Figure S6: $^1H$-$^{13}C$ HMBC NMR Spectrum (600 MHz, DMSO-d6, 298 K) for hesperetin 7'-O-α-D-glucopyranoside.**

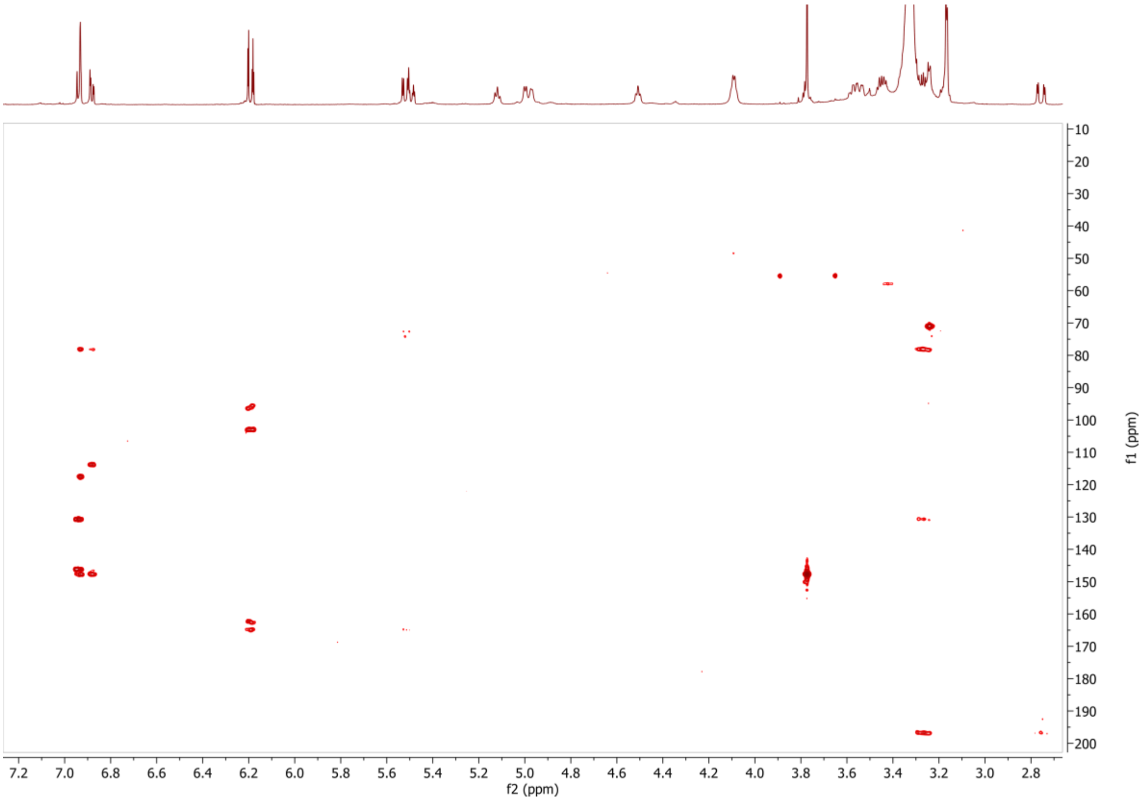


**Figure S7: Hesperetin (up) and hesperetin 7'-O-α-D-glucopyranoside signals affected by the diastereoisomers formation (down)**

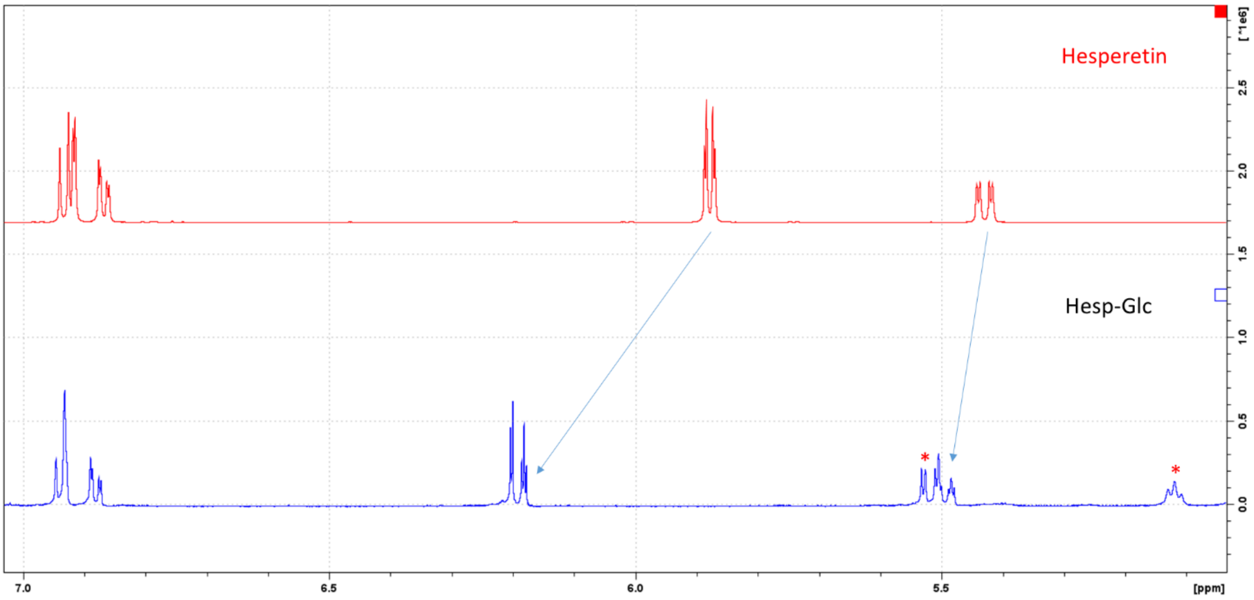

Supplement: Supplementary file 1 [file molecules-23-02885-s001.pdf]
